# Successful Coronary Artery Bypass Grafting in a Patient With Uncontrolled Scleromyxedema

**DOI:** 10.7759/cureus.7185

**Published:** 2020-03-05

**Authors:** Sohaip Kabashneh, Leo E Reap, Hammad Ali

**Affiliations:** 1 Internal Medicine, Wayne State University, Detroit Medical Center, Detroit, USA; 2 Hematology Oncology, Ascension Providence Hospital, Southfield, USA

**Keywords:** scleromyxedema, wound healing, systemic mucinosis

## Abstract

Scleromyxedema is a rare disorder characterized by diffuse cutaneous and systemic mucinosis with paraproteinemia. Affected patients usually develop numerous waxy, firm papules and plaques as a result of subcutaneous mucin deposition and fibrosis. Systemic manifestations may involve the cardiovascular, gastrointestinal, pulmonary, musculoskeletal, renal, or nervous systems and are known to lead to significant morbidity and mortality if left untreated. As the skin of these patients can be heavily infiltrated with mucin and fibrosis, it is unknown if scleromyxedema affects wound healing. Additionally, owing to the rarity of the disorder, there is very little data regarding surgical outcomes in these patients and their optimal management in the pre- and post-surgical setting. Herein, we report a case of a patient who underwent elective coronary artery bypass grafting (CABG) while in active relapse of his scleromyxedema; he suffered no pre- or post-operative complications and his surgical incision site healed well without any intervention.

## Introduction

Scleromyxedema is a rare disorder. It is characterized by diffuse cutaneous and systemic mucinosis with paraproteinemia. The definitive pathophysiology of why this occurs in the setting of a paraproteinemia remains unclear [1-2]. The diagnosis of scleromyxedema is based upon the recognition of the following clinicopathologic criteria: generalized, papular, and sclerodermoid eruption; microscopic triad, including mucin deposition, fibrosis, and fibroblast proliferation, or, less frequently, an interstitial granulomatous-like pattern; monoclonal gammopathy; absence of thyroid disorder.

Treatment for scleromyxedema remains fairly limited. As it affects the skin, wound healing in patients with scleromyxedema raises concerns when it comes to major surgeries. Our surgical experience with a patient with uncontrolled systemic scleromyxedema prompted this report.

## Case presentation

**Figure 1:**
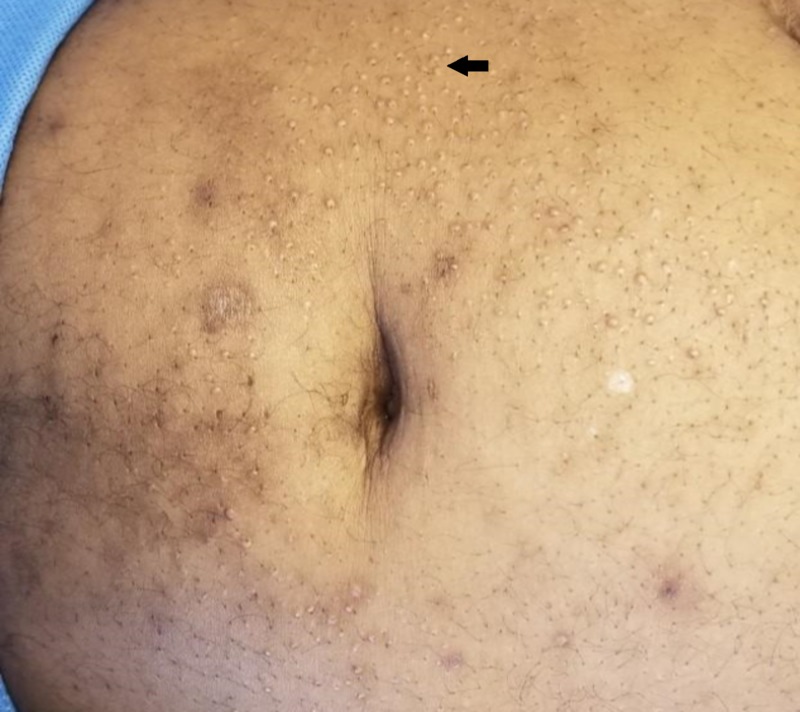
Numerous waxy nodules and plaques present across the ventral abdominal surface, characteristic of scleromyxedema

**Figure 2:**
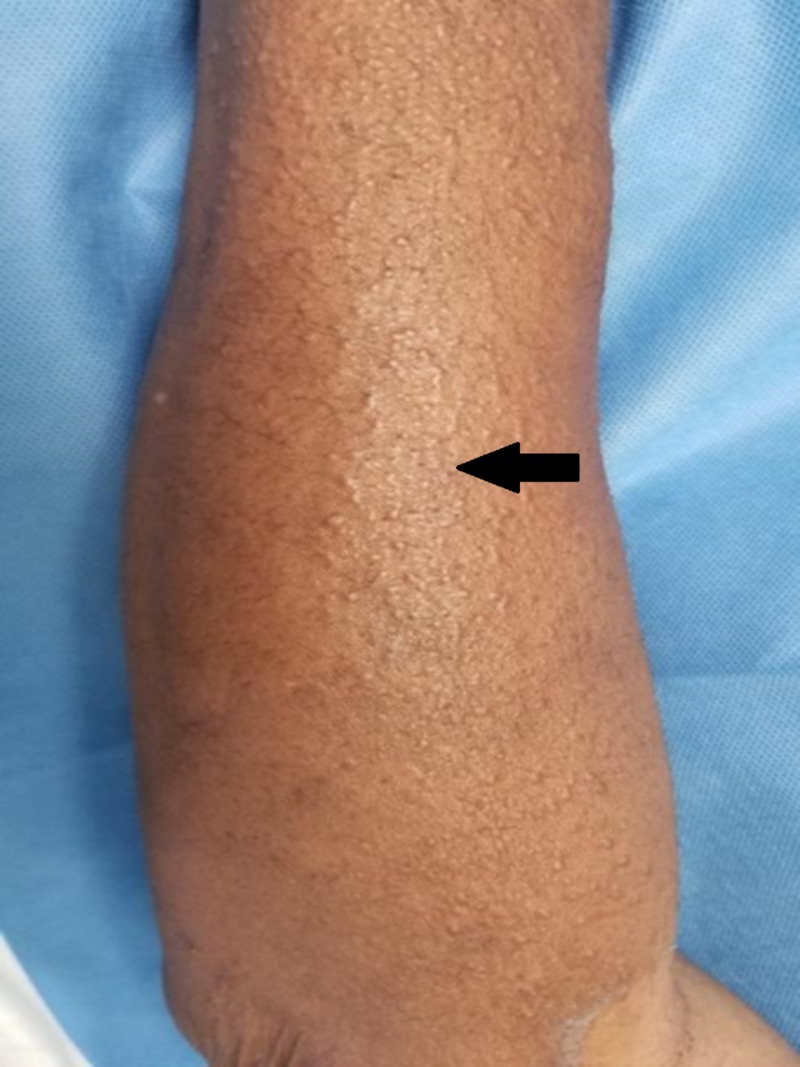
Innumerable waxy papules across the dorsal forearm, indicative of diffuse cutaneous involvement

**Figure 3:**
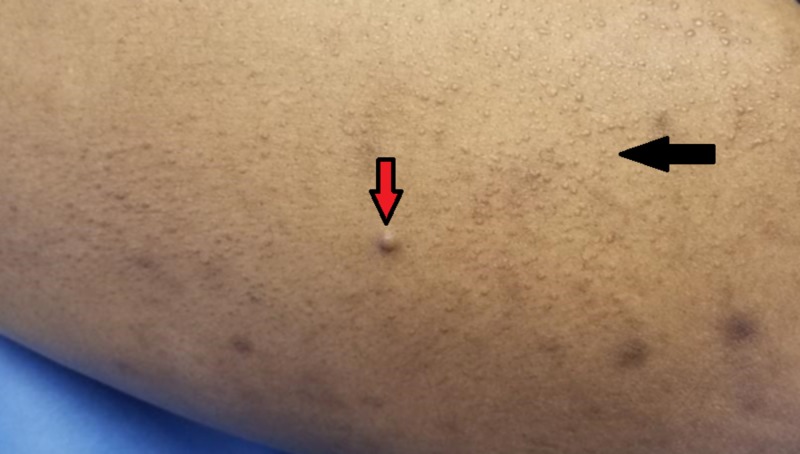
Scattered waxy papules and plaques across the ventral thigh (black arrow); a large subcutaneous nodule is also seen (red arrow)

**Figure 4:**
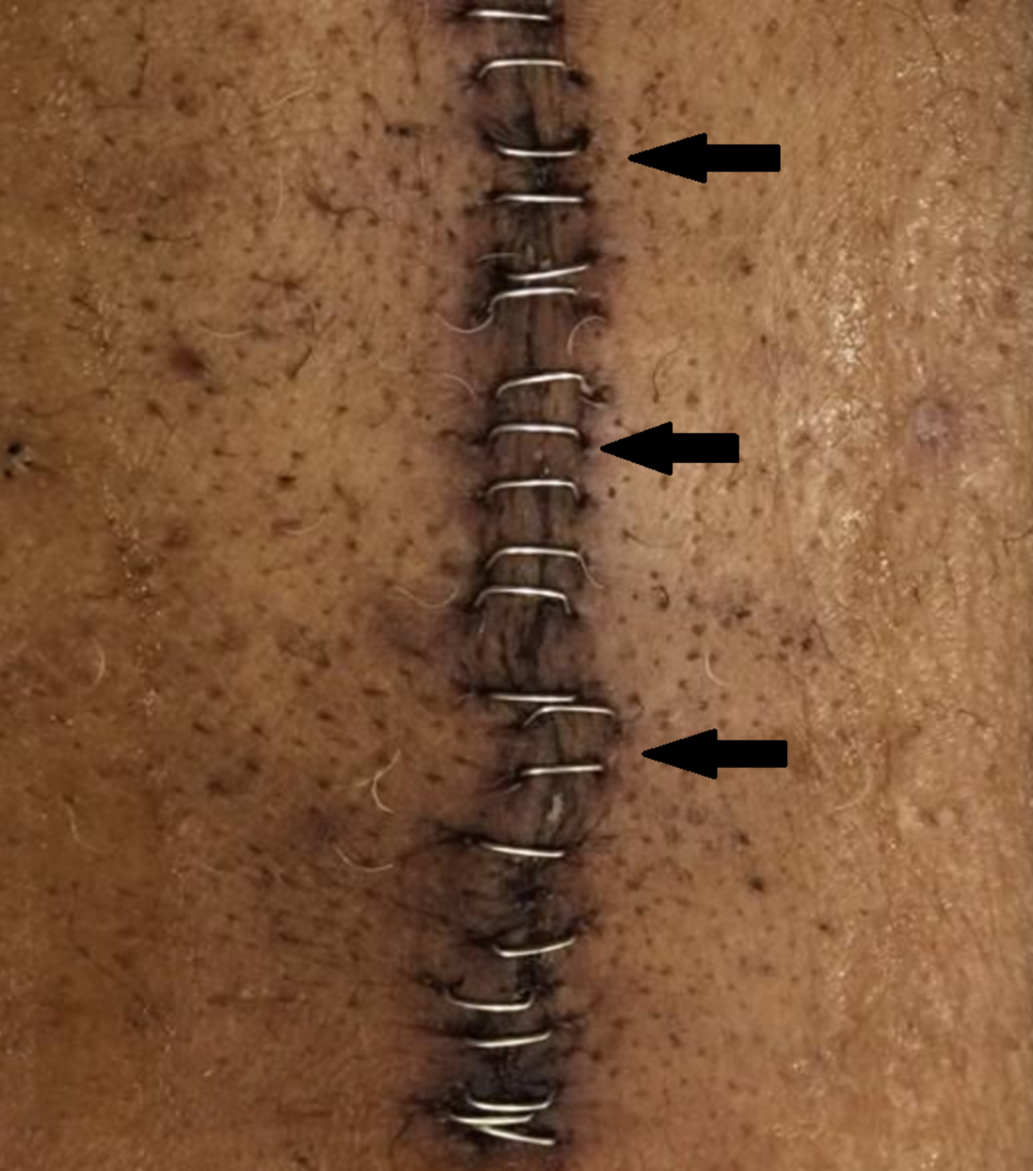
Post-operative chest incision on post-operative day seven displaying adequate wound healing

A 63-year-old man with known scleromyxedema presented to the hospital for elective coronary artery bypass grafting (CABG) but was discovered to have relapsed a few days prior to his scheduled surgery. He was diagnosed with scleromyxedema three years prior, manifesting as numerous waxy, firm papules and plaques involving his face, arms, and legs. Immunofixation demonstrated immunoglobulin G (IgG) lambda monoclonal gammopathy of 1040 mg/dL. Bone marrow biopsy revealed less than 5% lambda-restricted plasma cells. No evidence of CRAB (hypercalcemia, renal failure, anemia, bone disease) criteria were seen. Hemoglobin was 14 g/dl, calculated glomerular filtration rate (GFR) between 90 to 100 mL/min, corrected calcium of 9.3 mg/dl, and urinalysis demonstrated no evidence of proteinuria. Thyroid-stimulating hormone (TSH) was normal. Skin biopsy of the papules was histologically consistent with scleromyxedema. Monthly intravenous immunoglobulin (IVIg) was started in August 2017 with gradual improvement in his skin lesions, and complete resolution by December 2017. IgG lambda stabilized at 700-800 mg/dL and he remained asymptomatic, completing treatment in April 2018. However, one year later, his scleromyxedema relapsed with a gradual return of numerous cutaneous waxy papules and plaques (Figures 1-3). He did not follow up with dermatology and so was not resumed on treatment. In July 2019, he was discovered to have triple-vessel coronary artery disease after sustaining a myocardial infarction. Owing to concerns for increasing plasma viscosity and further limiting coronary artery blood flow, in addition to the amount of time required to attain treatment effect, IVIG was not able to be administered. Plasmapheresis was also not able to be performed due to the amount of time required to attain treatment effect. Corticosteroids were also avoided due to concerns for impairing surgical wound healing. As a result, he underwent CABG for triple vessel while in active relapse of his scleromyxedema. He suffered no complications postoperatively and his sternal incision site healed well (Figure 4).

## Discussion

Scleromyxedema is a rare disorder. The characteristic skin finding in scleromyxedema is a widespread appearance of waxy, firm, dome-shaped papules and plaques involving the face, extremities, chest, and abdomen. Papules are often arranged in a linear array. Rarely, nontender subcutaneous nodules are present. Deep, longitudinal furrows can be seen on the face, the trunk or limbs. Erythema, edema, and a brownish discoloration may be seen in the involved areas [1].

Infiltration of mucin and fibrosis into other organs, if left unchecked, leads to significant organ dysfunction. Almost any organ system may be involved, including the cardiovascular, gastrointestinal, pulmonary, musculoskeletal, renal, or nervous systems. Infiltration of cardiac tissue leads to increased rates of congestive heart failure, myocardial ischemia, heart block, and pericardial effusion [3-5].

Treatment for scleromyxedema remains fairly limited. Preferred initial therapy is IVIg which is based upon multiple case reports and case series that support its efficacy [6]. Plasmapheresis can be used to temporize the disorder, with scattered case reports displaying some efficacy [7]. In patients with an insufficient response to IVIG, systemic glucocorticoids and thalidomide have been used in patients with varying results [7-9].

Wound healing in patients with scleromyxedema raises concerns when it comes to major surgeries. There are only two cases reported in the literature about wound healing in patients with scleromyxedema subjected to a major surgical procedure. In 1946, a young woman had an abdominal hysterectomy, salpingo-oophorectomy, and appendectomy and healed without apparent difficulty [10]. The second case was in 1983, where a 46-year-old man with a poorly differentiated squamous-cell carcinoma of the bladder invading the rectosigmoid colon underwent a pelvic lymphadenectomy, radical cystectomy, and resection of the adjacent involved large bowel, with primary colorectal anastomosis and construction of a ureteroileal urinary conduit. The wound and all anastomoses also healed without apparent difficulty [10].

## Conclusions

Scleromyxedema is characterized by diffuse cutaneous and systemic mucinosis that can lead to infiltration of almost any organ. As the skin of these patients can be heavily infiltrated with mucin and fibrosis, wound healing in these patients raises concerns when it comes to major surgeries. In addition to the previous two case reports to date, our case suggests that although scleromyxedema leads to mucinous infiltration of the dermis, active disease does not appear to impair cutaneous or visceral wound healing ability. Patients with scleromyxedema appear to tolerate major surgery despite the severity of their skin manifestations.
